# Exploring Siglecs: Potential Modulators of Immune Cells in Food Allergy and Therapeutic Applications

**DOI:** 10.1111/cea.70119

**Published:** 2025-07-28

**Authors:** J. S. H. Schaapherder, K. C. M. Verhoeckx, A. M. Ehlers, E. F. Knol, A. C. Knulst, L. A. P. M. Meulenbroek

**Affiliations:** ^1^ University Medical Center Utrecht Utrecht the Netherlands; ^2^ Department of Chemical Biology and Drug Discovery, Utrecht Institute of Pharmaceutical Sciences Utrecht University Utrecht the Netherlands; ^3^ Danone Research & Innovation Utrecht the Netherlands

**Keywords:** food allergy, immune cells, inhibitory receptors, Siglecs

## Abstract

Food allergies (FAs) are common in society with limited treatment options available. Therefore, there is an urgent need for new targets and treatment options. Sialic acid‐binding immunoglobulin‐type lectins (Siglecs) are mostly inhibitory receptors differentially expressed on all immune cells. There are many different types of Siglecs, and they are involved in the regulation of several signalling pathways. The specific role that Siglecs can have on various diseases, including cancer and immune‐driven disorders, is gaining interest. This review focusses on the current knowledge of the role of Siglecs on immune cells involved in FA sensitisation and elicitation and how targeting these Siglecs could possibly prevent or treat FA. Most research is focussed on targeting Siglecs expressed by mast cells and basophils, and how this can dampen the activation and/or degranulation of these cells. Targeting Siglecs on cells involved in the sensitisation phase of FA could be an interesting option to intervene earlier on in the allergic response, thus preventing the onset of FA rather than treating it. Siglec‐2 on B cells is already of great interest for the treatment of FA, and results seem promising as B cell receptor signalling and antibody production were inhibited. Siglecs on other cell types in the sensitisation phase, such as dendritic cells, seem promising, but functional assays with human cells are lacking so far. Overall, Siglecs are broadly expressed on all immune cells involved in the allergic response, which support the hypothesis that Siglecs are involved in the allergic response itself and may act as a potential target in the treatment of FA. Especially, treatments focussed on targeting Siglecs on multiple immune cell types may have great potential, as this could enhance both efficacy and safety.

## Graphical abstract

Sialic acid‐binding immunoglobulin‐type lectins (Siglecs) are inhibitory receptors present on all immune cells involved in the allergic reaction. Currently, research is mostly focussed on targeting Siglecs on mast cells to possibly treat food allergies. However, Siglecs present on immune cells in the sensitisation phase of food allergy may also be interesting targets.

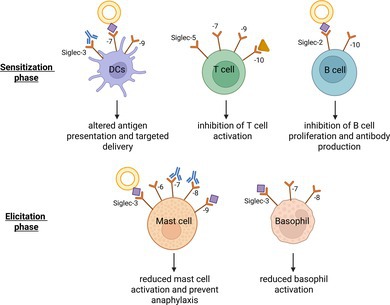

**Summary**

- Many immune cells show overlap in Siglec expression.
- Most research is focussed on targeting Siglecs on B cells, mast cells and basophils.
- Targeting Siglecs on multiple immune cells may enhance efficacy and safety.

## 1 Introduction

**FIGURE 1:**
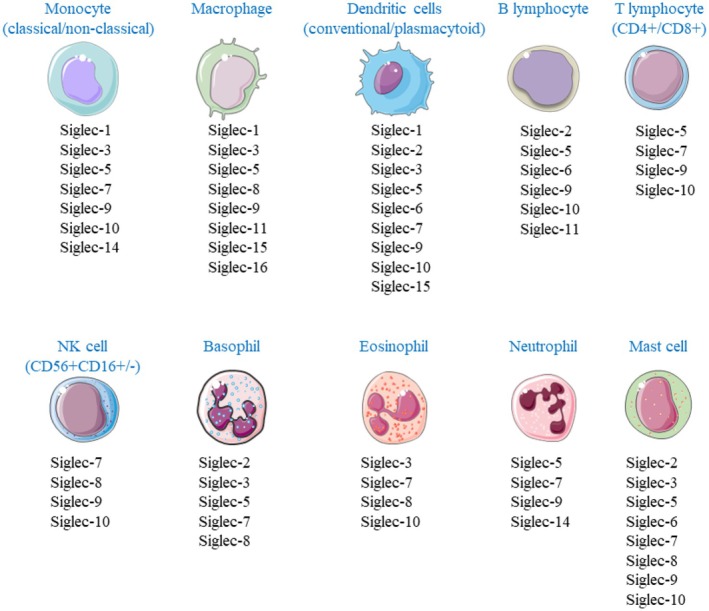
Overview of Siglec expression on immune cells [1, 2, 3, 4]. This figure is created using Servier Medical Art by Servier, licensed under Creative Commons Attribution 3.0 unported licence.

Currently, there is no cure for food allergy (FA) and there is a strong need for (new) treatment options. Sialic acid (SA)‐binding immunoglobulin‐type lectins (Siglecs) are mostly inhibitory receptors expressed on immune cells. In humans, 14 different Siglecs are expressed by haematopoietic cells (Figure 1). A positively charged arginine on the Siglec binds to the negatively charged carboxyl group of SA [1, 2, 3]. SAs are the terminal sugar moiety of glycans that are present on all human cell surfaces [4]. This enables our immune system to distinguish between self and non‐self. Siglec receptors can bind to SAs on the same cell (*cis*‐interactions) or to SA expressed on other cells or proteins (*trans*‐interactions). Intracellularly, most Siglecs contain an immune receptor tyrosine inhibitory motif (ITIM), allowing them to inhibit other cell processes [5, 6]. Upon binding of SA to these Siglecs, ITIM is phosphorylated, leading to the recruitment and binding of tyrosine phosphatases such as Src homology region 2 domain‐containing phosphatase (SHP)‐1 and SHP‐2, followed by inhibitory effects via dephosphorylation of other downstream targets of the Siglecs [7, 8, 9].

**FIGURE 2:**
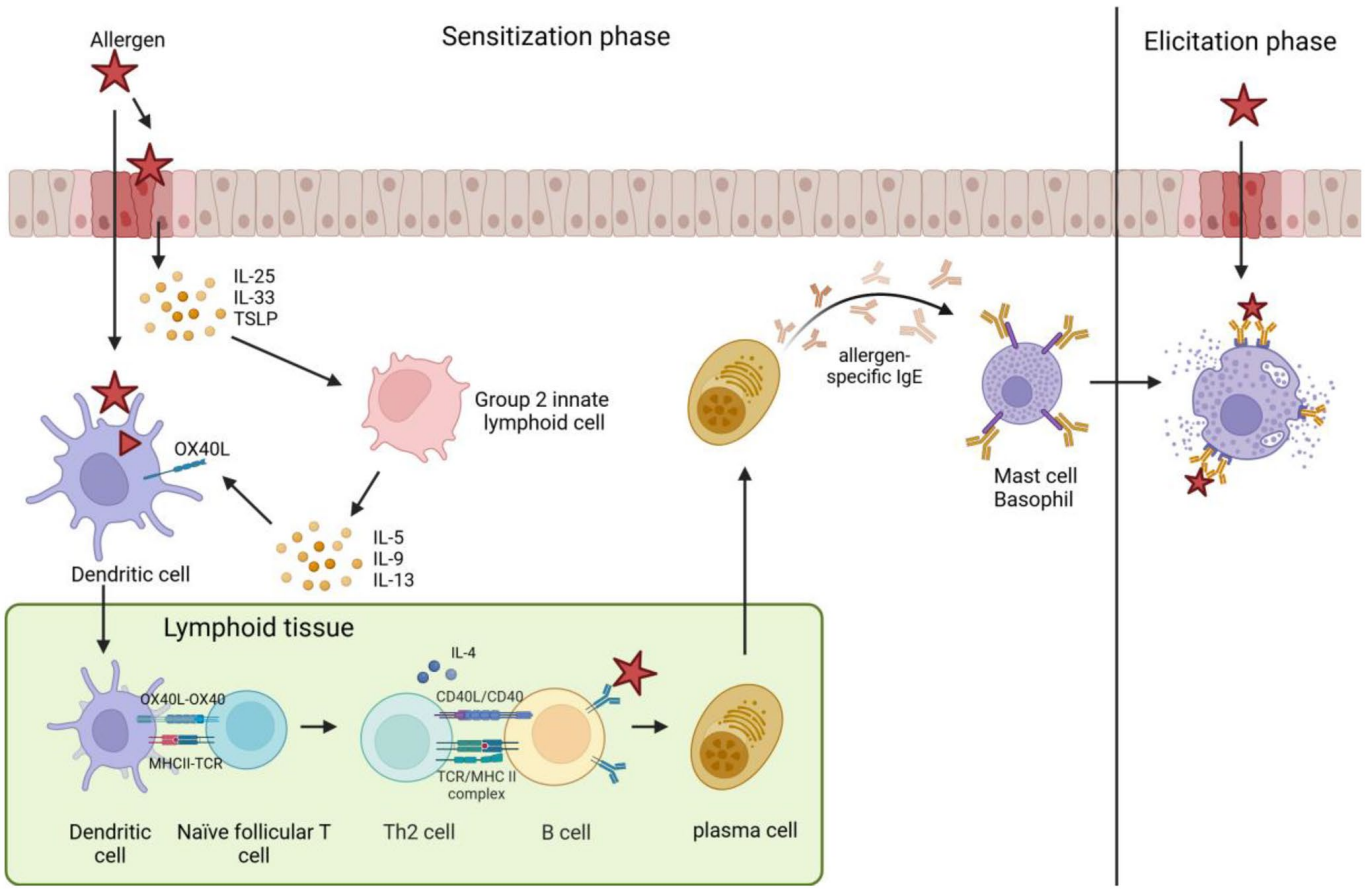
Simplified immunologic response underlying food allergy [5, 6]. Trans‐ or paracellular transport allows allergens to cross the epithelial barrier, accompanied by IL‐25, IL‐33 and TSLP release. This induces upregulation of OX40L in dendritic cells (DCs) and activation of group 2 innate lymphoid cells (ILC2s). Upon activation, ILC2s start to secrete cytokines (IL‐5, IL‐9 and IL‐13), which contribute to the Th2 response. DCs take up the allergen and migrate to the lymphoid tissue where they present processed peptides of the allergen via MHCII to naïve follicular T cells. In combination with OX40/OX40L interaction, this skews the T cell differentiation towards a Th2 response. These Th2 cells interact with allergen‐specific B cells via TCR/MHCII and CD40/CD40L, and in combination with allergen binding to the BCR and presentation by the B cell, this leads to the production of IL‐4 by Th2 cells and class switching of B cells into allergen‐specific IgE‐secreting plasma cells. The plasma cells migrate to the site of DC activation. IgE then binds to the high‐affinity FcεRI on effector cells such as mast cells and basophils. Upon a second allergen exposure, allergen binds FcεRI‐bound IgE, resulting in cross‐linking of FcεRI receptors. This initiates the release of allergic mediators from effector cells, resulting in the clinical outcome of food allergic reactions.

Siglecs have been shown to play an important role in various immune responses and diseases [6, 10]. This review focusses on the possible role of Siglecs in the immune cells involved in FA (Figure 2). Because many cell types are involved in FA and each cell has its own specific expression levels of Siglecs, we will discuss each cell type and the associated Siglecs on which functional research was performed separately.

## 2 Role of Siglecs in the Sensitisation Phase

In the following paragraphs, we describe the possible role of Siglecs in the sensitisation phase, except for type 2 innate lymphoid cells (ILC2s) for which no Siglec information was found. Where there is limited information about the role of Siglecs in FA, we instead focus on the known function of Siglecs and discuss their possible role in FA.

### 2.1 Dendritic Cells and Siglec‐3, ‐7, and ‐9

**FIGURE 3:**
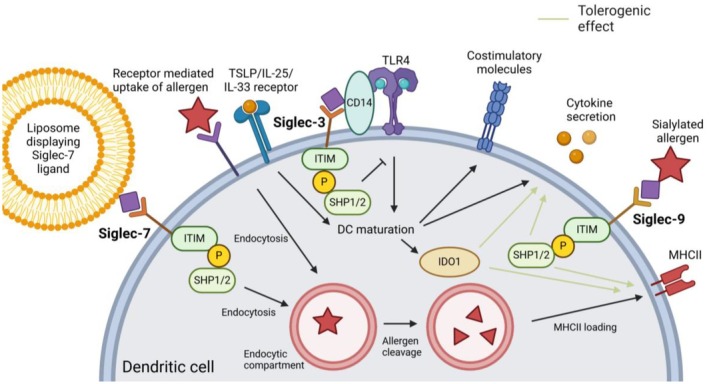
Effect of Siglec‐3, ‐7 and ‐9 on allergen presentation in dendritic cells. In food allergy, allergen is taken up, processed and presented on MHCII by DCs. Simultaneously, binding of cytokines (e.g., TSLP, IL‐25, IL‐33) results in the upregulation of costimulatory molecules and cytokine secretion. Siglec‐3 binding to CD14 may reduce DC maturation and survival, possibly via alteration of TLR4 signalling upon LPS binding. TLR4 is known to result in enhanced costimulatory molecule expression, important for DC differentiation, and cytokine secretion. In addition, IDO1 is secreted which may induce tolerance via altered cytokine secretion and altered allergen presentation. Siglec‐7 engagement by liposomes decorated with Siglec‐7 ligands results in endocytosis. The exact mechanism is unknown, but it may be of importance in the case of allergen presentation. Engagement of Siglec‐9 by an allergen expressing sialic acid may alter the allergic response in a more tolerogenic environment via cytokine secretion and altered T cell differentiation.

Dendritic cells (DCs) are key players in FA as the main allergen presenting cells (APCs). Upon allergen uptake, DCs process and present the allergen‐derived peptides on their major histocompatibility complex class II (MHCII) to CD4^+^ T cells, alongside DC maturation, enhanced expression of co‐stimulatory molecules, and cytokine secretion (Figure 3) [11, 12, 13, 14].

Siglecs may have an effect on these processes. Removal of surface SA enhanced DC maturation and antigen presenting capacity shown by an increased MHCII expression [15, 16, 17]. SA removal may disrupt *cis*‐interactions with Siglecs, thereby diminishing the inhibitory effect of Siglecs on the activation of DCs. Blockade of sialylation with a fluorinated SA mimetic diminished the interaction of SA with the Siglec receptors on DCs, resulting in increased responsiveness of DCs to Toll‐like receptor (TLR) activation and enhanced T cell activation in humans [18].

Siglecs also act as co‐receptors for other receptors like TLR4. Combined TLR4 and Siglec activation by LPS and α2,−3 SA in monocyte‐derived dendritic cells (moDCs) led to distinct activation of signalling molecules compared to activation of TLR4 or Siglecs alone [19]. This suggests a regulatory role of Siglecs on TLR4 signalling. The signalling molecules were mostly in the Janus kinase/signal transducer and activator of transcription (JAK/STAT) pathway, leading to increased IL‐10/IL‐12 ratio and thus reducing the inflammatory state. Whether TLR4 plays a role in FA is still under debate. Egg allergic children showed enhanced TLR4 activation in myeloid cells, and in the microbiota of allergic children, increased expression of genes involved in the biosynthesis of LPS was found [20, 21]. The effect of TLR4 activation can be broad and is dependent on the type and concentration of stimuli. For example, activation can lead to enhanced DC maturation and cytokine secretion, but also to the induction of tolerance [22, 23]. This makes it hard to speculate on its role in FA. The receptors of TSLP/IL‐25/IL‐33 also make use of the JAK/STAT signalling pathway, so Siglecs may also regulate these receptors [19, 24]. Unfortunately, to our knowledge, this has not been tested.

### 2.2 Siglec‐3

Immature DCs treated with an anti‐Siglec‐3 monoclonal antibody and LPS showed reduced phosphorylation of nuclear factor‐κB (NF‐κB) and subsequently reduced IL‐12 production, indicative of a lower inflammatory state [25]. The specific mechanism for this inhibitory effect of Siglec‐3 remains unclear as Siglec‐3 can bind to the sialylated chain of CD14, which is part of the TLR4 complex (Figure 3), but this was only to 2% of the total expressed CD14. Mature DCs were unresponsive to this Siglec‐3 engagement. As mature DCs lack CD14 expression, this does support the hypothesis of CD14 as a target of Siglec‐3. Additionally, anti‐Siglec‐3 treatment with a monoclonal antibody on CD14^+^ monocytes and CD34^+^ precursors resulted in reduced DC differentiation/maturation and survival, which again had no effect on mature DCs [26]. As the exact mechanisms of inhibition of TLR4 signalling by Siglec‐3 remain unknown, additional research is needed to elaborate on the effect of Siglec‐3 in DCs before its role in FA can be hypothesised.

### 2.3 Siglec‐7

The uptake of allergens can be affected by Siglecs, possibly altering allergen processing and presentation. Glycated β‐Lactoglobulin (BLG) showed higher binding efficiency to receptors on APCs (i.e., galectin‐3, CD36, and SR‐AI) leading to its internalisation [27]. Although this glycated BLG did not contain SA, it may be hypothesised that sialylated allergen uptake may also be enhanced due to their binding to Siglecs. This has been shown for Siglec‐7 on DCs. Liposomes decorated with Siglec‐7 ligands targeting moDCs induced uptake of their cargo by the moDCs (Figure 3) [28]. This indicated an endocytic function of Siglec‐7 on DCs. In established FA, modifying allergen uptake and presentation by DCs may be used in immunotherapy. Exposure to a low dose of allergens can lead to tolerance, and Siglec‐7 could be used as a target to deliver the allergen to APCs. In this way, allergen exposure could potentially be better controlled, hopefully leading to fewer side effects and promoting tolerance.

### 2.4 Siglec‐9

Siglec‐9 may inhibit TLR signalling in human moDCs [18]. Siglec‐9 is sensitive to *cis*‐interaction on DCs, which may inhibit TLR signalling via its ITIM, inducing a less activated phenotype in DCs (Figure 3). Siglec‐9 can also be targeted via *trans*‐interaction. Human moDCs were exposed to dendrimers targeting Siglec‐9 together with LPS [29], resulting in altered gene expression related to several processes including T cell differentiation. Functional analysis (e.g., cytokine analysis and T cell skewing) was indicative of a more tolerogenic T cell response.

This shift towards a tolerogenic T cell response seems promising and interesting to repeat in a FA setting using allergens instead of LPS. In mice, Siglec‐E (the mouse equivalent of Siglec‐9) has been studied in the context of allergen presentation by DCs. Allergens coupled to SA and targeting Siglec‐E were able to inhibit CD4^+^ and CD8^+^ T cell activation and promote the induction of allergen‐specific regulatory T cells in a non‐allergic setting, all via uptake by DCs [30]. Both results in human and murine DCs seem promising for Siglec‐9 as a DC target to induce tolerance in a food allergic setting.

### 2.5 T cells and Siglec‐5, ‐7, ‐9 and ‐10

**FIGURE 4:**
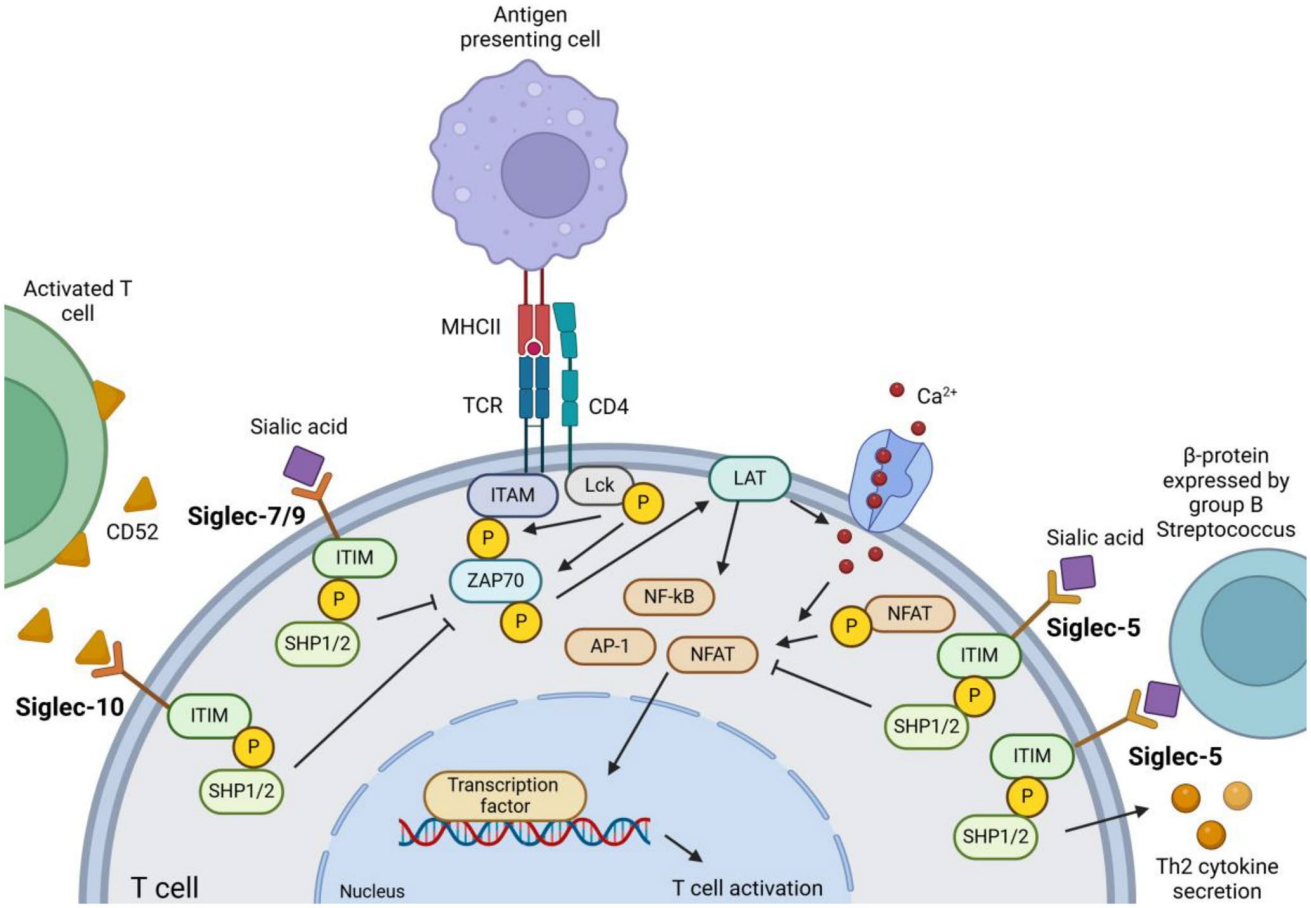
T cell activation regulated by Siglec‐5, ‐7, ‐9 and ‐10. Allergenic peptide presentation by MHCII to specific TCRs leads to the recruitment of CD4 to the TCR and subsequent phosphorylation of ITAM by Lck. ITAM phosphorylation leads to the recruitment of ZAP70, of which the phosphorylation is also mediated by Lck. ZAP70 in turn activates LAT, which serves as a docking station for other adaptor proteins, leading to the influx of calcium and the dephosphorylation of transcription factors like NF‐κB, AP‐1 and NFAT. These transcription factors translocate to the nucleus, where they can bind to DNA and upregulate the transcription of several cytokines and proteins involved in T cell activation. Overexpression of Siglec‐5 without adding a specific ligand leads to the inhibition of dephosphorylation of NFAT, thereby inhibiting T cell activation, whereas *trans* ligation of Siglec‐5 by β‐protein on group B Streptococcus enhances Th2 cytokine secretion. Siglec‐7 and Siglec‐9 inhibit ZAP70 activation, leading to further inhibition of the calcium influx and subsequent reduced T cell activation. Activated T cells express and secrete CD52. Soluble CD52 binds in a negative feedback loop to Siglec‐10, which also inhibits ZAP70 phosphorylation.

Presentation of allergenic peptides on MHCII by DCs to the T cell receptor (TCR) on allergen‐specific T cells results in TCR signalling (Figure 4) [31]. Together with the secreted cytokines by ILC2s (e.g., IL‐5/9/13) among others, this results in T cell differentiation and activation towards a Th2 phenotype, triggering sensitisation to food allergens [32].

### 2.6 Siglec‐5

Siglec‐5 plays a role in both CD4^+^ and CD8^+^ T cell activation. Upregulation of Siglec‐5 expression was detected on activated CD4^+^ and CD8^+^ T cells [33]. Overexpression of Siglec‐5 on Jurkat T cells inhibited TCR signalling by inhibiting the activity of nuclear factor of activated T cells (NFAT) and activating protein‐1 (AP‐1) after stimulation with anti‐CD3 or Phorbol 12‐myristate 13‐acetate/Ionomycin. This effect was seen without addition of a Siglec‐5 ligand, suggesting that Siglec‐5 ligands can be present on the same T cell (*cis*‐binding) or neighbouring T cell (*trans*‐binding). When using a specific Siglec‐5 ligand (β‐protein) expressed by group B Streptococcus, inflammatory cytokine secretion was inhibited while Th2 cytokine secretion was slightly enhanced (Figure 4). So, while *cis*‐interactions may lead to reduced T cell activation by inhibiting TCR signalling, *trans*‐ligation may promote a Th2 response. Because Siglec‐5 expression is activation dependent and different ligands can have different outcomes on T cell differentiation, Siglec‐5 seems a less suitable target for FA treatment.

### 2.7 Siglec‐7 and Siglec‐9

Expression of Siglec‐7 and Siglec‐9 on T cells is activation independent and minimal expression of these Siglecs is shown on resting T cells [33]. Siglec‐7 is expressed on a subset of CD8^+^ T cells, while Siglec‐9 is expressed on subsets of both CD4^+^ and CD8^+^ T cells. Expression has been shown to be individual‐dependent [34]. To study the effect of Siglec‐7 and Siglec‐9 on TCR signalling, Jurkat T cells were transfected with these Siglecs. Both Siglec‐7 and Siglec‐9 were co‐localised with the TCR, and upon TCR activation, the Siglecs recruited and activated SHP‐1, which led to reduced zeta‐chain‐associated protein kinase 70 (ZAP‐70) phosphorylation and inhibition of transcription factors like NFAT (Figure 4). Siglec‐9 was more effective in inhibiting T cell activation than Siglec‐7. No specific Siglec ligands were used, but an intact binding pocket of the Siglecs was crucial for this inhibitory effect. It was not clear whether this involved *cis‐* and/or *trans‐*interactions between the Siglecs and its ligands.

In FA, allergens are presented to naive CD4^+^ T cells; therefore, Siglec‐9 could be an interesting target to prevent TCR activation and further stimulation of B cells by T cells, thereby reducing the allergic response. However, the effect of Siglec‐9 on cytokine production has not yet been established. Similar to Siglec‐5, it is possible that Siglec‐9 ligation can lead to enhanced Th2 cytokine production and thus promote FA development.

### 2.8 Siglec‐10

Siglec‐10 is expressed on CD4^+^ T cells and its expression was enhanced upon T cell activation together with the expression of the glycoprotein CD52 [35]. Activated T cells can secrete soluble CD52, which binds to Siglec‐10, resulting in inhibitory signalling indicated by reduced expression of activation markers (CD69 and CD25) and reduced proliferation upon CD3/CD28 stimulation [36]. Binding of CD52 to Siglec‐10 leads to inhibition of tyrosine‐protein kinase Lck and ZAP70 phosphorylation, which are part of the TCR signalling cascade (Figure 4). In this way, Siglec‐10 mediates a negative feedback loop regulating T cell activation.

The specific role of Siglec‐10 on T cells in an allergic setting is unknown, but CD52 has shown to play a role in autoimmune diseases. Diabetic patients had a lower prevalence of CD52^hi^CD4^+^ T cells, thereby lacking suppressor activity, possibly via Siglec‐10 [36]. A comparable mechanism could apply to FA because also here an unwanted immune response towards a harmless protein is seen. If Siglec‐10 can be targeted with a synthetic ligand, this may induce inhibitory signalling in T cells, even when CD52 is lacking.

### 2.9 B cells and Siglec‐2 and ‐10

**FIGURE 5:**
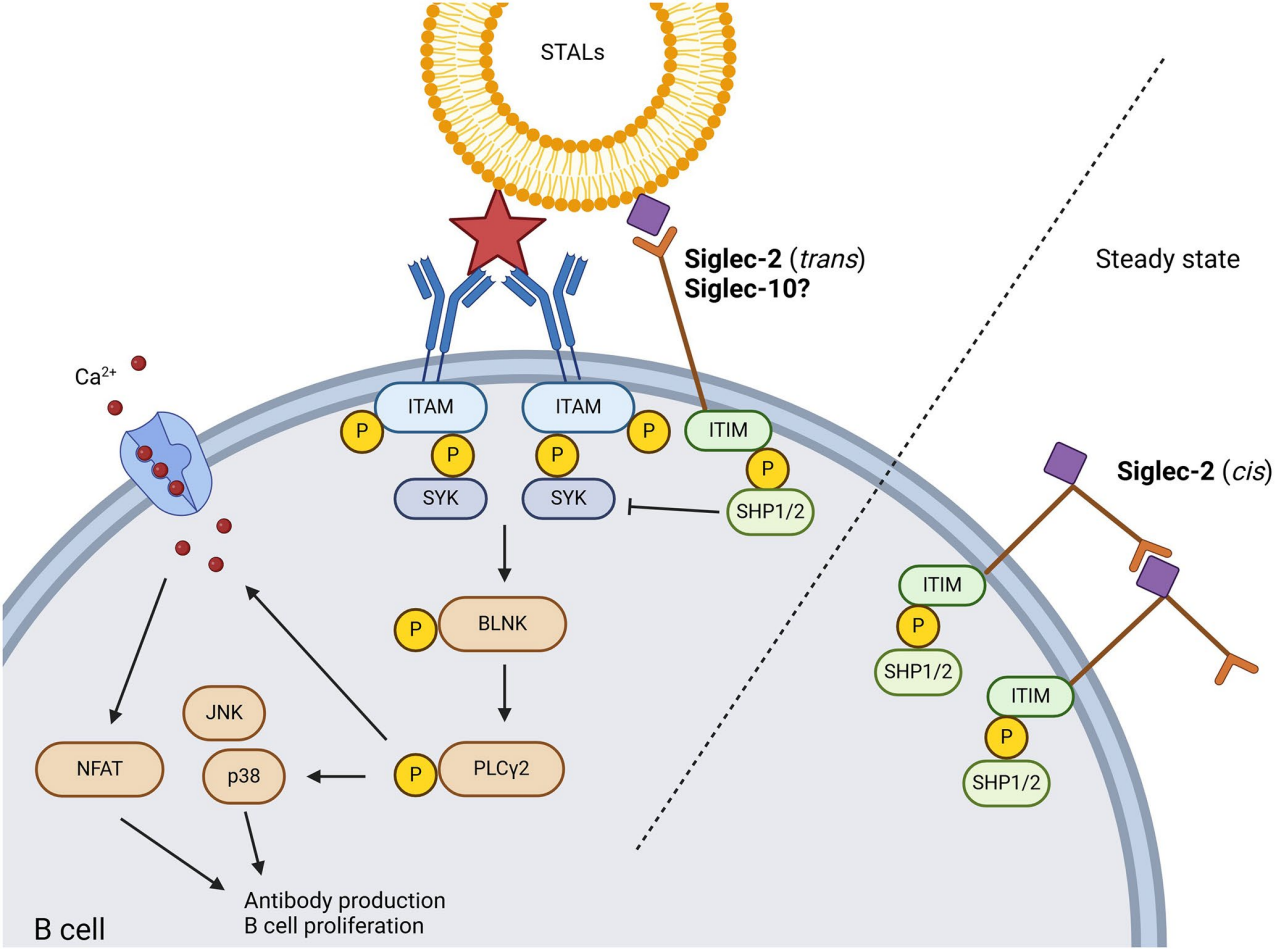
BCR signalling is regulated by Siglec‐2 in B cells. At steady state, Siglec‐2 is anchored by *cis*‐interactions with neighbouring Siglec‐2 receptors. BCR signalling is initiated when an allergen is bound by the BCR and crosslinking is induced. This results in phosphorylation of ITAM, which in turn recruits SYK to this complex. SYK is important for the activation of BLNK and subsequently PLCγ2. This initiates the influx of calcium, leading to the activation of transcription factor NFAT. Besides regulating the calcium influx, BLNK also regulates JNK and p38 activation. Eventually, all these proteins lead to plasma cell differentiation. The BCR can also interact with CD19, leading to the PI3k/IRF4/AKT/mTOR pathway. This also results in plasma cell differentiation. Siglec‐engaging tolerance‐inducing antigenic liposomes (STALs) carrying both allergen and Siglec‐2 ligands induce co‐localisation of Siglec‐2 and the BCR, resulting in inhibition of SYK and CD19 pathway and inhibits B cell proliferation and antibody production. Siglec‐10 may act in a similar way as Siglec‐2, but this has not been shown yet.

B cells play an important role in allergy because their activation by B cell receptor (BCR) ligation and Th2 cells induces class‐switching to IgE^+^ B cells and subsequent production of allergen‐specific IgE (Figure 5) [37].

### 2.10 Siglec‐2

Siglec‐2 is an inhibitory receptor anchored by *cis*‐interactions with neighbouring Siglec‐2 receptors on the same cell (Figure 5) [38]. Siglec‐2 is known to regulate the activation of several receptors on B cells (e.g., BCR). Different stimuli and methods to target Siglec‐2 resulted in contradictory outcomes. For example, Siglec‐2^−/−^ B cells treated with anti‐IgM impaired proliferation compared to wild‐type B cells [39]. This proliferative effect of Siglec‐2 was also seen when *cis‐*interactions of Siglec‐2 were disrupted in wild‐type B cells by an anti‐Siglec‐2 antibody. This effect was also seen with the use of synthetic Siglec‐2 ligands [40]. This study showed enhanced proliferation by adding a combination of anti‐CD40, anti‐IgM and Siglec‐2 ligand. Overall, *cis*‐interaction may regulate B cell activation by direct co‐ligation, which may be disrupted by *trans*‐interaction. This would explain why co‐cross‐linking of Siglec‐2 and the BCR is needed for inhibitory targeting in *trans*, as will be discussed next.

Siglec‐2 on B cells was targeted in various immunomodulating studies. Polymers carrying both an allergen and Siglec‐2 ligands inhibited BCR activation, demonstrated by inhibition of SYK phosphorylation and calcium influx [41]. The importance of co‐crosslinking Siglec‐2 and BCR was underscored by the lack of inhibition in the presence of non‐crosslinking reagents. In other studies, Siglec‐engaging tolerance‐inducing allergenic liposomes (STALs), that is, liposomal nanoparticles decorated with both Siglec ligands and an allergen, were used (Figure 5). Reduced allergen‐specific IgE (sIgE) levels were found in mice treated with STALs carrying BPA‐Neu5Gc (Siglec‐2 ligand) and peanut allergen Ara h 2 before sensitisation with whole peanut extract [42]. Moreover, it reduced sIgE to peanut and Ara h 1, and attenuated anaphylaxis upon peanut extract administration. STALs displaying peanut allergens Ara h 1, 2 or 3 and ligands for Siglec‐2 were used to target Siglec‐2 on memory B cells in a splenocyte transfer mouse model [43]. This treatment resulted in prolonged tolerance towards Ara h 1, 2 and 3. Additionally, STALs containing BPA‐Neu5Gc and hen's egg lysozyme (HEL) induced an allergen‐specific tolerogenic effect, indicated by reduced sIgE and apoptosis induction in mouse HEL‐reactive B cells via hypophosphorylation of spleen‐associated tyrosine kinase (SYK), CD19 and downstream targets (Figure 5) [44]. They also targeted Siglec‐2 on human B cells with STALs decorated with anti‐IgM and anti‐IgG antibodies, which led to induction of apoptosis. However, no allergic model was applied. Hardy et al. did focus on a mouse model where the B cells expressed human Siglec‐2 [43]. B cells were targeted with STALs displaying Siglec‐2 ligands and Ara h 1, which resulted in lower IgG titers compared to mice targeted with STALs displaying only Ara h 1. Unfortunately, functional assays such as measuring the anaphylactic potential after allergen challenge are lacking.

Overall STALs show promise for treating FA. However, more studies are needed to demonstrate their efficacy and safety on human B cells. So far, most studies were performed in mice or not in an allergy‐specific setting, and it is unknown whether the obtained results can be translated to humans.

### 2.11 Siglec‐10

Siglec‐10 is expressed on the majority of B cells [45], but functional studies on Siglec‐10 in allergic or immunodeficient settings have not been performed. Siglec‐10 is not conserved between humans and mice, and the mouse equivalent of Siglec‐10 is Siglec‐G. Ligation of Siglec‐G on B cells using a liposome carrying a specific Siglec‐G ligand and HEL inhibited B cell activation and induced tolerance in a similar manner as shown for Siglec‐2 [46]. It is not clear how to translate the findings in mice to humans.

## 3 Role of Siglecs in the Elicitation Phase

The effector phase in allergy is characterised by clinical symptoms caused by the mediator release from mast cells and basophils (Figure 2). Because much is known about the role of Siglecs on these cells, we will limit the information on the general function and focus more on their role in the allergic reaction.

### 3.1 Mast cells and Siglec‐3, ‐6, ‐7, ‐8, and ‐9

**FIGURE 6:**
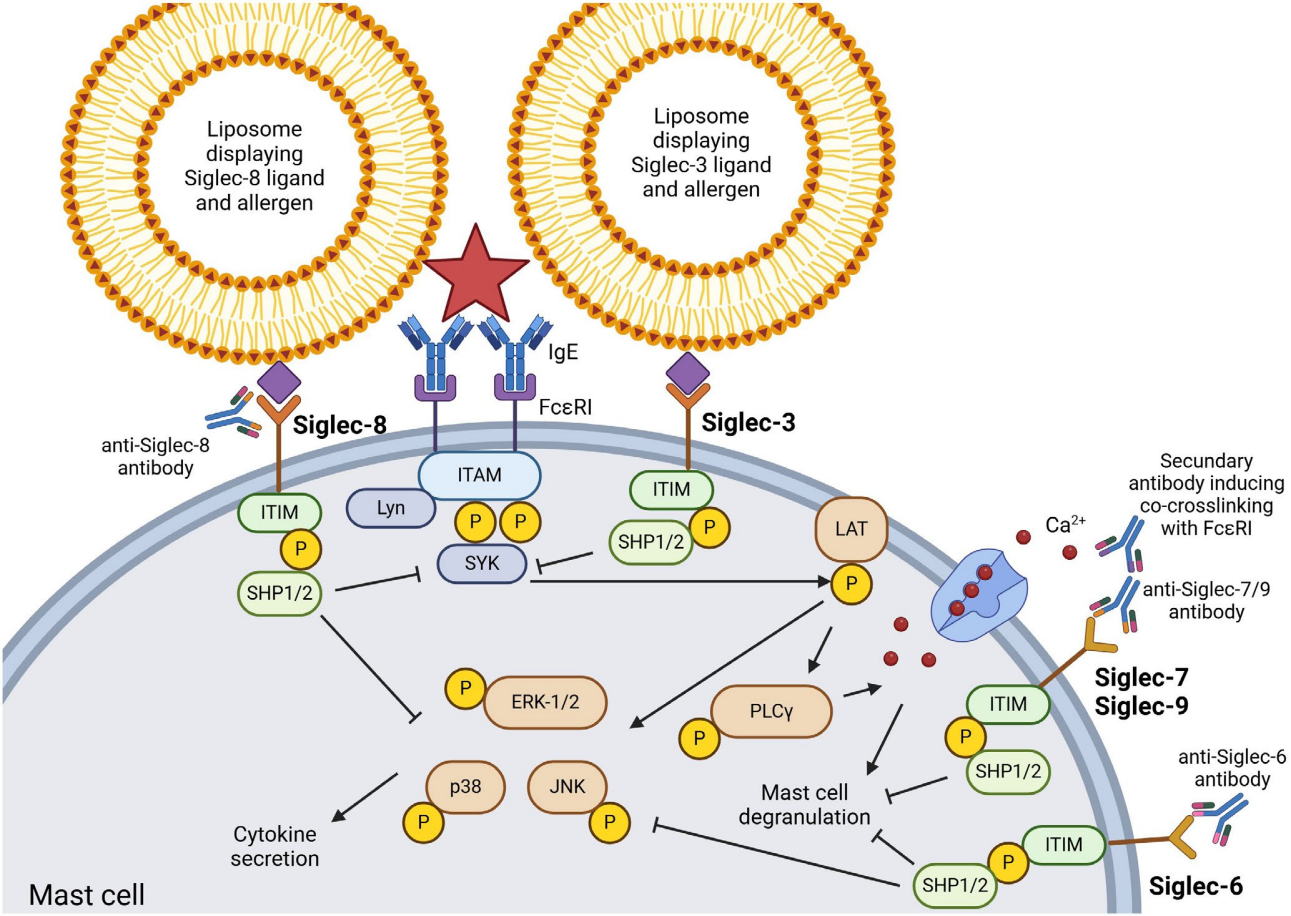
Simplified overview of FcεRI signalling in mast cells influenced by Siglecs. For better visualisation, the signalling cascade of only one FcεRI is displayed. Upon FcεRI crosslinking with an allergen, ITAM in the FcεRI gets phosphorylated by Lyn, leading to the recruitment of SYK. SYK, in turn, leads to the activation of the membrane protein LAT. Activation of LAT initiates phosphorylation of ERK‐1/2, p38 and JNK, which are all involved in the synthesis and secretion of cytokines. LAT can also phosphorylate PLCγ, which initiates the influx of calcium, leading to mast cell degranulation. Siglec‐3 can inhibit mast cell degranulation after co‐crosslinking with FcεRI via dephosphorylation of several kinases in the FcεRI signalling cascade by SHP1/2. Siglec‐6 can inhibit FcεRI signalling by inhibiting the phosphorylation of ERK‐1/2 and p38, inhibiting cytokine secretion and can directly inhibit mast cell degranulation. Co‐crosslinking with FcεRI is not needed for inhibition, but it can enhance inhibition. Siglec‐7 and Siglec‐9 inhibit FcεRI signalling, yet the mechanisms remain unelucidated. Co‐crosslinking with the FcεRI was essential for the inhibitory effect. Siglec‐8 can inhibit both mast cell degranulation and cytokine secretion with and without co‐crosslinking with the FcεRI by inhibiting the FcεRI complex, SYK and downstream targets PLCγ, ERK‐1/2, p38 and JNK.

Mast cell activation due to crosslinking of the high‐affinity IgE receptor (FcεRI) is characterised by degranulation of mast cells, releasing allergic mediators such as histamine and cytokines. The FcεRI signalling cascade has been extensively reviewed by others (Figure 6) [47, 48, 49].

### 3.2 Siglec‐3

The function of Siglec‐3 on mast cells at steady state is unknown, but Siglec‐3 can be targeted to diminish mast cell activity [50]. Liposomes displaying a high‐affinity ligand for Siglec‐3 and Ara h 2 inhibited mast cell degranulation in the human mast cell line LAD2. No inhibition was seen with a mixture of liposomes bearing the ligand or allergen alone, indicating the need for co‐crosslinking of the FcεRI and the Siglec receptor. In a humanised Siglec‐3 mouse model, liposomes with ovalbumin and a Siglec‐3 ligand inhibited mast cells and prevented thereby anaphylaxis up to 24 h after liposome administration, while liposomes with either ligand or allergen alone could not.

Islam et al. coupled a Siglec‐3 ligand directly to an anti‐IgE antibody, which was also able to reduce mast cell activation ex vivo and to prevent anaphylaxis in a humanised mice model [51]. It has been proposed that recruitment of Siglec‐3 to the FcεRI‐complex leads to phosphorylation of the ITIM‐domains of Siglec‐3 by local Src kinases and results in the recruitment of SHP‐1 (Figure 6) [50]. SHP‐1 can then dephosphorylate several kinases involved in the FcεRI‐signalling cascade, leading to inhibition of this cascade. Taken together, these results suggest that Siglec‐3 seems a suitable target to diminish mast cell degranulation in FA.

### 3.3 Siglec‐6

Siglec‐6 was previously considered a mast cell‐specific Siglec, although its expression has now also been reported on B cells and DCs. In human skin‐derived mast cells, Siglec‐6 engagement by a monoclonal antibody reduced the β‐hexosaminidase release when mast cells were exposed to IgE‐dependent and IgE‐independent mechanisms [52]. This effect was even prolonged as mast cell stimulation was inhibited 4.5 h after Siglec‐6 antibody administration. By co‐crosslinking Siglec‐6 and the FcεRI complex with a secondary antibody, the inhibitory effect on mast cells was enhanced. This was shown by the reduced expression of the degranulation markers CD63 and lysosomal‐associated membrane protein 1 (LAMP1), lower release of allergic mediators (tryptase and β‐hexosaminidase) and cytokines (macrophage inflammatory protein‐1β, IL‐8, IL‐1β). The inhibition was mediated by reduced phosphorylation of extracellular signal‐regulated kinases (ERK)‐1/2 and p38, which are both downstream targets of FcεRI (Figure 6) [53]. Internalisation of Siglec‐6 was shown to be slower than for Siglec‐8, another mast cell Siglec. This may explain the prolonged effect of Siglec‐6 compared to Siglec‐8. Korver et al. also showed a stronger effect of Siglec‐6 compared to Siglec‐8, despite similar expression levels [54].

The fact that co‐crosslinking of Siglec‐6 with the FcεRI is not required to inhibit mast cells favours the use of Siglec‐6 as a target in FA treatment. Moreover, the prolonged effects of Siglec‐6 engagement are promising [52]. However, the findings in vitro still need to be translated to the in vivo situation.

### 3.4 Siglec‐7

Siglec‐7 also has an inhibitory effect on mast cells. When activated using a monoclonal antibody and co‐crosslinked with the FcεRI‐complex, mast cell activation was inhibited as shown by decreased release of allergic mediators (tryptase and β‐hexosaminidase) and the cytokine GM‐CSF (Figure 6) [55]. Co‐crosslinking is essential for the inhibitory role of Siglec‐7, as it did not show this effect when no co‐crosslinking was induced. The mechanism via which Siglec‐7 is functioning remains unclear, as SHP‐1 phosphorylation could not fully explain the inhibitory effect of Siglec‐7. Siglec‐7 ligand coupled to the allergen could be used to prevent FA symptoms when the inhibitory effect of Siglec‐7 is strong enough in an in vivo allergic setting, but this needs to be elucidated.

### 3.5 Siglec‐8

Siglec‐8 is expressed by both tissue‐derived and cultured mast cells and is involved in the regulation of mast cell activation and degranulation [56, 57]. This is demonstrated by agonistic anti‐Siglec‐8 monoclonal antibodies. Only preincubation with these antibodies before FcεRI crosslinking inhibited the cytosolic calcium increase and subsequently reduced the histamine release by 50% in human mast cell cultures [58]. Siglec‐8 interacts with multiple proteins involved in FcεRI signalling, like the gamma subunit of FcεRI itself and Lyn, and subunits of cytokine receptors, leading to the inhibition of mast cell activation [54, 59].

Different studies investigated how Siglec‐8 can be targeted to treat mast cell‐mediated diseases. One study reported on a Siglec‐8 humanised monoclonal antibody AK002 (lirentelimab), which is being investigated in clinical trials for treatment of several mast cell‐ and eosinophil‐mediated diseases [60, 61]. Lirentelimab was well tolerated in these patients and showed clinical improvement of symptoms. To elucidate the mechanism of lirentelimab, experiments have been performed with the mouse precursors of lirentelimab, mAK003. Anaphylaxis was inhibited in humanised mice by administering the antibody 24 h before passive sensitisation [62]. Culturing ex vivo dissociated lung tissue overnight with lirentelimab showed no reduction in mast cell numbers, suggesting that the inhibition was due to reduced mast cell activation rather than mast cell apoptosis. This was mediated via inhibition of kinases and signalling molecules involved in the FcεRI signalling pathway (e.g., FcεRI, SYK, and ERK) (Figure 6) [63, 64].

Besides the use of antibodies, liposomes decorated with Siglec‐8 ligands and allergens (trinitrophenol or ovalbumin) are also in development [65, 66]. These liposomes inhibited degranulation of mast cells in a humanised mouse model by dephosphorylation of kinases involved in calcium mobilisation (e.g., phospholipase C (PLC)γ) and cytokine transcription and secretion (e.g., ERK‐1/2, p38, and JNK) (Figure 6). Moreover, they were able to prevent anaphylaxis during a challenge with ovalbumin, demonstrating the therapeutic relevance of these liposomes in FA [65]. Even 5 h after liposome injection, mice were still protected from anaphylaxis. Duan et al. showed that murine mast cells injected with these liposomes were unable to bind the allergen due to binding of the liposome to the IgE‐FcεRI complex or due to internalisation of the FcεRI. Altogether, these results show desensitisation of mast cells in these mice. Interestingly, co‐crosslinking of Siglec‐8 to the IgE‐FcεRI complex was necessary in this setting, which is in contrast with Siglec‐8 engagement with the antibody lirentelimab [62, 64]. One explanation might be steric hindrance between FcεRI and Siglec‐8 by separately decorated liposomes as they are much larger than an antibody alone. Duan et al. also proposed that the FcεRI activation induced by liposomes may be much stronger than by single allergens in solution due to increased avidity, meaning that stronger inhibition may be required.

Overall, Siglec‐8 has proven to be a suitable target for other mast cell‐mediated diseases in humans and for FA in mice. On human mast cells, antibodies directed against Siglec‐8 are promising and favourable as no co‐crosslinking with the FcεRI needs to be induced. Therefore, lirentelimab may also be suitable for the treatment of FA.

### 3.6 Siglec‐9

Recently, Siglec‐9 expression was discovered on mast cell lines and human mast cells [67]. Incubation of LAD2 with natural Siglec‐9 ligands (GlycA and HMW‐HA) in combination with an IgE‐dependent or IgE‐independent stimulus resulted in reduced release of β‐hexosaminidase. Interestingly, in primary human mast cells, co‐crosslinking of a Siglec‐9 monoclonal antibody and the FcεRI was needed to achieve inhibition of mast cell activation. Siglec‐9 deficient LAD2 cells showed increased expression of LAMP‐1 (activation marker) and were more susceptible to IgE‐dependent and IgE‐independent activation. Together, this suggests that under normal circumstances Siglec‐9 is bound by *cis*‐interactions which dampen the activation of mast cells.

Despite the fact that the mechanism of action of Siglec‐9 in mast cells remains unknown, the effect on mast cells is the same as for the other expressed Siglecs, namely inhibition of mast cell degranulation. Therefore, Siglec‐9 may be a target for the treatment of FA. Miralda et al. already indicated that the addition of Siglec‐9 as a possible target on mast cells is of importance since the function of Siglecs on mast cells may differ between tissues or may change over time [67]. For instance, skin‐derived mast cells showed higher inhibition of mast cell activation upon Siglec‐9 ligation than peripheral blood cultured mast cells.

### 3.7 Basophils and Siglec‐3, ‐7 and ‐8

Basophils are the least abundant granulocytes but play an important role in FA [68]. Basophils are blood‐dwelling cells that can migrate to (chronically) inflamed tissues where they secrete allergic mediators and cytokines, such as histamine and IL‐4, upon cross‐linking of the IgE–FcεRI complex by allergens. The FcεRI signalling in these cells is similar to the signalling pathway in mast cells (Figure 6). Since basophils and mast cells show overlap with expressed Siglecs, some compounds that were tested on mast cells have also been tested on basophils (e.g., Siglec‐3 ligands, Siglec‐7 antibodies, Siglec‐8 liposomes).

### 3.8 Siglec‐3

The general function of Siglec‐3 on basophils remains unknown. Similar to mast cells, Siglec‐3 on basophils can be targeted by a complex of a Siglec‐3 ligand coupled to anti‐IgE to reduce basophil activation [51, 69]. Siglec‐3 was higher expressed on basophils than Siglec‐8, which will be discussed later. The higher expression of Siglec‐3 is promising, as it may result in a higher potency for basophil inhibition. Treatment of passively sensitised basophils as well as basophils from peanut allergic donors with the anti‐IgE–Siglec‐3 ligand complex before stimulation with whole peanut extract indeed led to reduced basophil activation. The mechanism responsible for this inhibition and the clinical application of this complex are unknown.

### 3.9 Siglec‐7

Despite the fact that both mast cells and basophils express Siglec‐7, co‐crosslinking of Siglec‐7 with the FcεRI on basophils with monoclonal antibodies was not as effective in inhibiting histamine release as it was on mast cells [55]. Mizrahi et al. suggested that the difference in inhibiting capacity of Siglec‐7 is due to a difference in downstream signalling rather than its expression levels, which were comparable on both cells. Unfortunately, they did not investigate the Siglec‐7 signalling cascade in basophils. As the inhibitory effect of Siglec‐7 on basophils was only minor, it is unlikely that Siglec‐7 is a suitable target in FA.

### 3.10 Siglec‐8

Siglec‐8 expression is much lower on basophils than on mast cells. However, its function is similar: inhibition of mast cell/basophil activation. Unfortunately, the basophil inhibition mechanisms by Siglec‐8 targeting have not been investigated. As the outcome is comparable to those in mast cells, we may assume that Siglec‐8 may also interact with components of the FcεRI signalling cascade in basophils.

Unfortunately, not much Siglec‐8‐targeted therapy has been tested on basophils. Lirentelimab, the anti‐Siglec‐8 monoclonal antibody, showed minimal binding to Siglec‐8 on basophils, which may not be a surprise due to its low expression [61]. Therefore, Siglec‐8 on basophils is probably a less suitable target for FA treatment than Siglec‐8 on mast cells.

## 4 Concluding Remarks

Siglecs are highly abundant on all immune cells and are known to influence signalling pathways in many diseases. Therefore, Siglecs may be potential targets to control immune responses. The role of Siglecs in various diseases is not yet fully understood. This is especially true for FA. Only a few studies have focussed on Siglecs in FA. Overall, these studies showed an inhibitory role of Siglecs on immune cell activation. The experimental conditions may not always be specific for FA, but the fact that similar pathways were activated as upon allergen stimulation makes it likely that the results can be extrapolated to a food allergic setting. Most research related to the treatment of FA and Siglecs focusses on the effector phase and B cells (e.g., targeting Siglec‐6 and Siglec‐8 on mast cells or STALs‐targeting Siglec‐2 on B cells). This is in line with the current strategy in the treatment of FA, which is based on treating the symptoms rather than preventing the allergic sensitisation. Targeting immune cells involved in the sensitisation phase (e.g., DCs and T cells) may be a better strategy to reduce (re‐)sensitisation and the eventual development of FA. Siglecs on these cells may therefore be an interesting target to prevent the (re‐)sensitisation to food allergens. For instance, Siglec‐9 on both DCs and T cells could be an interesting target for this purpose as it has a potential inhibitory role on both cell types. The fact that Siglec‐9 is also expressed on mast cells makes it an even more promising target, as both immune cells in the sensitisation phase and elicitation phase can be targeted with one ligand. This combination of targeting Siglecs on immune cells in the sensitisation and elicitation phase simultaneously potentially enhances the therapeutic potential and/or safety of these treatments. In addition, one could also think about combining Siglec‐targeted treatments directed against B and T cells, to possibly enhance or prolong the inhibitory effects seen on isolated B and T cells.

Overall, more research is needed on the specific functioning of Siglecs on human immune cells and their potential as targets in a food allergic setting.

## Author Contributions

J.S.H. Schaapherder, K.C.M. Verhoeckx and L.A.P.M. Meulenbroek designed the review and drafted the manuscript. A.M. Ehlers, E.F. Knol and A.C. Knulst reviewed and commented on the manuscript. All authors approved the final manuscript.

## Conflicts of Interest

L.M. is employed by Danone Research & Innovation. A.E. has a guest appointment at Danone Research & Innovation. A.K. has received institutional sponsoring for research or consultancy from ALK‐Abelló A/S, Thermo Fisher Scientific, Nutricia/Danone, DBV technologies, Novartis, EUROIMMUN, Stallergenes Greer, TNO, FARRP, NVWA and STW. J.S., K.V. and E.K. have no conflicts of interest to declare.

## Data Availability

Data sharing not applicable to this article as no datasets were generated or analysed during the current study.
